# Factors associated with changes in psychological resilience of older adults with mild cognitive impairment during the COVID-19 pandemic

**DOI:** 10.3389/fnagi.2023.1169891

**Published:** 2023-08-11

**Authors:** Nanae Matsumoto, Yujiro Kuroda, Taiki Sugimoto, Kosuke Fujita, Kazuaki Uchida, Yoshinobu Kishino, Hidenori Arai, Takashi Sakurai

**Affiliations:** ^1^Department of Prevention and Care Science, National Center for Geriatrics and Gerontology, Research Institute, Obu, Aichi, Japan; ^2^Center for Comprehensive Care and Research on Memory Disorders, National Center for Geriatrics and Gerontology, Obu, Aichi, Japan; ^3^Department of Rehabilitation Science, Kobe University Graduate School of Health Sciences, Kobe, Hyogo, Japan; ^4^Department of Cognitive and Behavioral Science, Nagoya University Graduate School of Medicine, Nagoya, Aichi, Japan; ^5^National Center for Geriatrics and Gerontology, Obu, Aichi, Japan

**Keywords:** older adults, mild cognitive impairment, psychological resilience, CD-RISC-10, COVID-19, sleep quality

## Abstract

**Introduction:**

Psychological resilience is an indicator of mental health, but there has been no research to date on changes in psychological resilience among older adults with mild cognitive impairment (MCI) during the stress of the coronavirus disease 2019 (COVID-19) pandemic including factors related to those changes. To fill the gap, this study examined the factors and the changes in psychological resilience of older adults with MCI during the COVID-19 pandemic.

**Methods:**

One hundred thirty participants completed the 10-item version of the Connor-Davidson Resilience Scale (CD-RISC-10) between December 2020 and June 2021 as a baseline assessment and between December 2021 and February 2022 as a follow-up. Participants also answered questions on sleep quality, depression symptoms, activities in daily living (ADL), instrumental ADL and social participation to explore factors associated with changes.

**Results:**

In this cohort, the mean CD-RISC-10 scores were significantly higher than the baseline at follow-up (*p* < 0.05), indicating the improvement of psychological resilience. In multiple regression analyses, sleep quality was significantly correlated with change in CD-RISC-10 score (coefficient = 3.94, 95% confidence interval = 1.11 to 6.78).

**Discussion:**

Psychological resilience could improve even during the stress of the COVID-19 pandemic in older adults with MCI who were at risk of developing dementia. The factor associated with improved psychological resilience was good sleep quality.

## 1. Introduction

The coronavirus disease 2019 (COVID-19) pandemic began spreading in Japan in March 2020, and the Japanese government declared a state of emergency in April 2020 to mitigate the infection spread. People were strongly recommended to stay at home and were restricted from using facilities where people gather; public life faced social restrictions to enforce infection control measures. Research on mental health among older adults with mild cognitive impairment (MCI) during the COVID-19 pandemic is important because a large number of older adults with MCI or at risk of dementia developed increased anxiety and depression during the lockdown (Barguilla et al., 2020) and faced severe adversity. Taking preventive measures against mental health problems among older adults with MCI in the future is thus important.

Psychological resilience is the ability to recover one’s mental health following adversity or stressful experiences (Levasseur et al., 2017). This acts as a protective factor preventing the worsening of depressive symptoms (Chang et al., 2023a). Some studies have reported that several brain regions are involved in psychological resilience, including the medial prefrontal cortex (mPFC), which functions as a mechanism for psychological resilience (Bremner, 2007; Menon and Uddin, 2010; Hsieh et al., 2021; Chang et al., 2023b). However, the state of psychological resilience in older adults with MCI has not been described; understanding this state can help develop supportive mental health measures. Therefore, we previously examined the psychological resilience of older adults with MCI during the COVID-19 pandemic in a cross-sectional study using the Japanese version 10-item version of the Connor-Davidson Resilience Scale (CD-RISC-10), which has confirmed reliability and validity (Campbell-Sills and Stein, 2007; Ito et al., 2009). In our previous study, it was reported that the psychological resilience of the participants was observed to be low (Matsumoto et al., 2022). However, other studies have found that psychological resilience increases with exercise and treatment (Pakalniskiene et al., 2016; Eyre et al., 2017). In other words, psychological resilience may be a dynamic factor. In addition, psychological resilience improves with time more than depression and anxiety indicators (Okuyama et al., 2018). Thus, our hypothesis was that psychological resilience can improve in older adults with MCI. Therefore, we investigated changes in the psychological resilience of older adults with MCI during the COVID-19 pandemic. Further, we explored factors associated with the change in psychological resilience. Investigating changes in psychological resilience in older adults with MCI and the factors associated with such changes can be expected to contribute to the development of more specific supportive interventions to help prevent mental health deterioration in the face of natural disasters or the spread of new infectious diseases.

## 2. Methods

### 2.1. Study design

We conducted a longitudinal study during the COVID-19 pandemic as a substudy of the Japan-Multimodal Intervention Trial for Prevention of Dementia (J-MINT) (Sugimoto et al., 2021). We conducted the CD-RISC-10 as a baseline from December 2020 to June 2021 with a follow-up from December 2021 to February 2022. We conducted assessments other than CD-RISC-10 between February 2020 and March 2021.

### 2.2. Participants

The target participants were 150 older adults with MCI aged 65–85 years in the control group of J-MINT, where we considered a diagnosis of MCI if they fulfilled the following criteria: (1) having a cognitive decline in the National Center for Geriatrics and Gerontology (NCGG) Functional Assessment Tool (Makizako et al., 2013; Shimada et al., 2017), (2) Mini-Mental State Examination (MMSE) (Folstein et al., 1975) score of 24 or more at baseline, and (3) not diagnosed with dementia. A cognitive decline in NCGG-FAT is age- and education-adjusted cognitive decline with a standard deviation (SD) of ≥1.0 from the reference threshold on at least one of the four cognitive domains of memory, attention, executive function, and processing speed as measured by the NCGG-FAT. Participants with self-reported depression or missing data on the CD-RISC-10 were excluded. We conducted the study with the approval of the NCGG Ethics Committee (No. 1468).

### 2.3. Measurements

We administered a questionnaire to the participants that collected information on sociodemographic characteristics (age, sex, marital status, education, and household income) as well as the Barthel Index (Mahoney and Barthel, 1965), Lawton Index (Lawton and Brody, 1969), CD-RISC-10, 15-item Geriatric Depression Scale (GDS) (Sugishita et al., 2017), Pittsburgh Sleep Quality Index (PSQI) (Buysse et al., 1989), and social participation scores (Kanamori et al., 2014). A Barthel Index score of 100 indicates complete independence. Perfect scores of Lawton Index are 5 for men and 8 for women, it was treated as a binary value with a perfect score and no perfect score. A CD-RISC-10 score ranges is from 0 to 40, and higher CD-RISC-10 scores reflect greater psychological resilience. The reliability of the CD-RISC-10 was moderate among the older adults, with a mean score of MMSE 27.2 (Tourunen et al., 2021). A GDS score ranges is from 0 to 15, and the score of 7 or more indicates depressive symptoms and was treated as a binary value. A PSQI score ranges is from 0 to 21, and the score of 5 or less indicates good sleep quality and was treated as a binary value. We measured social participation by asking participants if they belonged to any of eight types of organizations listed, with each organization type scored as 1 point.

### 2.4. Statistical analysis

We calculated means, standard deviations, medians, interquartile ranges, frequencies, and percentages to describe the demographic data in the participants as appropriate. We then used a paired-sample *t*-test to compare the baseline and follow-up CD-RISC-10 scores. We further used single and multivariate analyses to evaluate the relationships between changes in the CD-RISC-10 score (CD-RISC-10 values at follow-up minus values at baseline) and explanatory variables. To develop a multivariate model, we included as moderator variables sociodemographic characteristics, which have been associated with psychological resilience in previous studies (Campbell-Sills et al., 2009; Matsumoto et al., 2022). Then, we performed multiple regression analysis with the change in the CD-RISC-10 score as the objective variable and assessments other than CD-RISC-10, MMSE, and baseline CD-RISC-10 score as explanatory variables, thereby obtaining regression coefficients and 95% confidence intervals. We performed all analyses using Stata 16.1 (Stata Corp, College Station, TX, USA), considering *p* < 0.05 statistically significant.

## 3. Results

We received 136 responses from the 150 participants who received our questionnaire (90.7%). We excluded 6 participants who met the exclusion criteria, resulting in 130 participants. The mean age of the study participants was 73.1 ± 4.5 years; 42.3% were female; 80.0% were married; the mean number of years of education was 12.5 ± 2.3 (Table 1). The mean CD-RISC-10 scores were 24.1 ± 6.3 and 26.6 ± 7.8 points at the baseline and the follow-up, respectively (*p* = 0.0001), for a median change of 3 points. In the single regression analysis, the only factor that was significantly associated with the change in the CD-RISC-10 score was the baseline CD-RISC-10 score (coefficient = −0.35, 95% CI = −0.54 to −0.17); the PSQI tended to be associated, but not statistically significant (coefficient = 2.86, 95% CI = −0.01 to 5.73) (Table 2). In contrast, in multivariate models with all explanatory variables entered simultaneously, both the PSQI score and the baseline CD-RISC-10 score were significantly associated with changes in the CD-RISC-10 score (coefficient = 3.94, 95% CI = 1.11 to 6.78 and coefficient = −0.37, 95% CI = −0.56 to −0.18, respectively).

**TABLE 1:** Demographic characteristics of participants (*n* = 130).

Attribute information	All participants
Age, mean ± SD	73.1 ± 4.5
Female, *n* (%)	55 (42.3)
Marital status: married, *n* (%)	104 (80.0)
Education years, mean ± SD	12.5 ± 2.3
Household income, *n* (%)	
<JPY 2,000,000	16 (12.3)
JPY 2,000,000–3,990,000	65 (50.0)
JPY 4,000,000–5,990,000	25 (19.2)
JPY 6,000,000–7,990,000	13 (10.0)
JPY 8,000,000–9,990,000	9 (6.9)
JPY 10,000,000 and above	2 (1.5)
NCGG-FAT: cognitive decline, *n* (%)	
Memory	55 (42.3)
Attention	52 (40.0)
Executive function	41 (31.5)
Processing speed	4 (3.1)
Barthel Index, median (IQR)	100 (100, 100)
Lawton Index: expect a perfect score, *n* (%)	7 (5.4)
MMSE, median (IQR)	28 (26, 29)
Social participation, mean ± SD	1.2 ± 0.9
PSQI: good sleep quality, *n* (%)	100 (76.9)
GDS: depressive symptoms, *n* (%)	12 (9.2)
CD-RISC-10: baseline, median (IQR)	24 (20, 27)
CD-RISC-10: follow-up, median (IQR)	26 (21, 33)

CD-RISC-10, 10-item Connor–Davidson Resilience Scale; GDS, 15-item Geriatric Depression Scale; IQR, interquartile range; JPY, Japanese Yen; MMSE, Mini-Mental State Examination; NCGG-FAT, National Center for Geriatrics and Gerontology Functional Assessment Tool; PSQI, Pittsburgh Sleep Quality Index.

**TABLE 2:** Simple and multiple regression analysis results for changes in psychological resilience.

	Simple regression analysis		Multiple regression analysis	
	Coefficient	95% CI	Coefficient	95% CI
Age	-0.26	−0.53 to 0.01	-0.22	−0.48 to 0.05
Sex: female	-1.28	−3.76 to 1.19	-0.82	−3.29 to 1.64
Marital status: married	1.31	−1.75 to 4.37	-0.21	−3.32 to 2.91
Education	-0.23	−0.77 to 0.31	-0.49	−1.01 to 0.04
Household income	-0.87	−1.94 to 0.19	-1.07	−2.12 to −0.01
Barthel Index	1.25	−0.00 to 2.51	1.03	−0.19 to 2.25
Lawton Index: no perfect score	0.15	−5.29 to 5.59	0.27	−4.90 to 5.43
MMSE	0.53	−0.17 to 1.23	0.28	−0.44 to 0.99
Social participation	0.16	−0.75 to 1.08	0.50	−0.36 to 1.36
PSQI: good sleep quality	2.86	−0.01 to 5.73	3.94	1.11 to 6.78*
GDS: no depressive symptoms	0.38	−3.86 to 4.62	1.35	−2.82 to 5.51
CD-RISC-10	-0.35	−0.54 to −0.17*	-0.37	−0.56 to −0.18*

*p < 0.05.

CD-RISC-10, 10-item Connor–Davidson Resilience Scale; CI, confidence interval; GDS-15, 15-item Geriatric Depression Scale; MMSE, Mini-Mental State Examination; PSQI, Pittsburgh Sleep Quality Index.

## 4. Discussion

We investigated changes in psychological resilience in older adults with MCI during the COVID-19 restrictions; interestingly, the results showed a trend toward increased psychological resilience. In addition, we investigated the associations between changes in CD-RISC-10 score and factors that have been related to psychological resilience (Aburn et al., 2016; Clement-Carbonell et al., 2019; Bazzani et al., 2021). Among the associated factors, the PSQI score was positively associated with changes in the CD-RISC-10 score; good sleep quality was a factor that may be associated with an increase in psychological resilience.

It is necessary to understand the state of psychological resilience in this vulnerable population so that intervention measures are already in place in the event of future disasters or other emergencies. The results of our investigations could help in developing preventive measures against mental health deterioration in this population.

In a previous examination of the psychological resilience of adolescents affected by a natural disaster, the young people’s median CD-RISC-10 score increased from 20 to 23 in 1 year (Okuyama et al., 2018), similar to the change in the CD-RISC-10 score observed in this study, although we cannot directly compare the change in CD-RISC-10 score from the present study with the change from the previous study. Our findings indicated that older adults with MCI can improve their psychological resilience after some time even if they also have a risk of developing dementia. In addition, this study was conducted during the COVID-19 pandemic, and the results indicate that psychological resilience improves even in the presence of stress due to, for instance, social contact restrictions. However, the social restrictions differed at baseline and follow-up, and stress was not constant: a state of emergency was announced at baseline, and semi-emergency coronavirus measures were applied in follow-up. This stress reduction may be associated with increased psychological resilience.

Good sleep quality was a factor associated with changes in psychological resilience. Psychological resilience entails high control of one’s emotions under stress (Bazzani et al., 2021), and poor sleep quality can impair this emotional functioning (Walker, 2009). In other words, psychological resilience might have improved in the present study because respondents who experienced good sleep quality were able to maintain high functioning in their emotion control spheres. In neuroimaging, psychological resilience is related to the frontal-associated regions, including the mPFC, amygdala, and anterior cingulate cortex (Hsieh et al., 2021). Among these frontal-associated regions, the functional connectivity between the mPFC and the amygdala is involved in sleep and emotion. The mPFC modulates the amygdala, which processes emotions (Walker, 2009). In previous research, sleep-deprived people have exhibited significant amygdala activation in response to negative stimuli, as well as a significant loss of functional connectivity in contrast with a control group (Walker, 2009). Sleep deprivation may impair the functional connectivity of the mPFC and amygdala, reducing the psychological resilience involving these regions. However, this apparent connection between psychological resilience and sleep quality remains speculative.

This present study has some limitations. First, Aichi and Gifu prefectures, where the study participants live, were placed under a state of emergency from mid-January to February 2021 (during the baseline), and participants responded to the questionnaires in different infection statuses, but we did not include these differences in our analyses. Second, our findings have limited generalizability to broader populations, because this is a substudy of the J-MINT and we did not perform random sampling. Third, the COVID-19 pandemic has been ongoing in Japan since March 2020, and people have faced social restrictions for a long time. Due to long-term social restrictions, people may have adjusted to restricted lifestyles, increasing their psychological resilience. Our analyses did not reflect this factor. Fourth, it was not possible to determine whether changes in psychological resilience differed by type of cognitive impairment, as the present study did not distinguish participants by type. Fifth, this study was unable to determine why resilience improved because sleep quality and depressive symptoms were not assessed with the follow-up in this study.

This study showed that psychological resilience improved after some time even during the stress of the COVID-19 pandemic in a population of older adults with MCI who were at risk of declining mental health. For instance, good sleep quality was related to improvements in psychological resilience, which maintains mental health. However, further research may be needed to determine whether factors related to changes in psychological resilience contribute to the maintenance of mental health in older adults with MCI.

## Data availability statement

The original contributions presented in the study are included in the article, further inquiries can be directed to the corresponding author.

## Ethics statement

The studies involving human participants were reviewed and approved by the National Center for Geriatrics and Gerontology Ethics Committee (No. 1468). The patients/participants provided their written informed consent to participate in this study.

## Author contributions

NM designed the study, performed the statistical analyses, and wrote the first draft. All authors contributed to the interpretation and discussion of the results and reviewed the manuscript and contributed to the article and approved the submitted version.
